# Classification of Transgenic Mice by Retinal Imaging Using SVMS

**DOI:** 10.1155/2022/9063880

**Published:** 2022-06-29

**Authors:** Farrukh Sayeed, K. Rafeeq Ahmed, M. S. Vinmathi, A. Indira Priyadarsini, Charles Babu Gundupalli, Vikas Tripathi, Wesam Shishah, Venkatesa Prabhu Sundramurthy

**Affiliations:** ^1^Department of Electrical and Electronics Engineering, ACE College of Engineering, Trivandrum 695027, Kerala, India; ^2^Department of Electronics and Communication Engineering, School of Engineering, Presidency University, Bangalore 560064, India; ^3^Department of Computer Science & Engineering, Panimalar Engineering College, Chennai 600123, Tamil Nadu, India; ^4^Department of Botany, SKR Govt. Degree College, Nagari, Andhra Pradesh 517590, India; ^5^Department of Computer Science & Engineering, Gokaraju Rangaraju Institute of Engineering and Technology (Autonomous), Kukatpally, Telangana 500090, India; ^6^Department of Computer Science & Engineering, Graphic Era Deemed to Be University, Dehradun, Uttarakhand 248002, India; ^7^College of Computing and Informatics, Saudi Electronic University, Riyadh, Saudi Arabia; ^8^Department of Chemical Engineering, Addis Ababa Science and Technology University, Addis Ababa, Ethiopia

## Abstract

Alzheimer's disease is the neuro disorder which characterized by means of Amyloid– *β* (A  *β*) in brain. However, accurate detection of this disease is a challenging task since the pathological issues of brain are complex in identification. In this paper, the changes associated with the retinal imaging for Alzheimer's disease are classified into two classes such as wild-type (WT) and transgenic mice model (TMM). For testing, optical coherence tomography (OCT) images are used to classify into two groups. The classification is implemented by support vector machines with the optimum kernel selection using a genetic algorithm. Among several kernel functions of SVM, the radial basis kernel function provides the better classification result. In order to deal with an effective classification using SVM, texture features of retinal images are extracted and selected. The overall accuracy reached 92% and 91% of precision for the classification of transgenic mice.

## 1. Introduction

**Figure 1:**
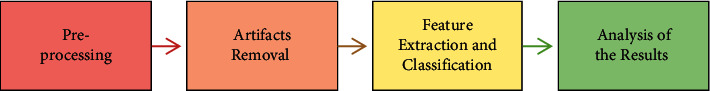
Typical flow for transgenic mice.

The most common form of disability is neurodegenerative disease [1, 2]. Because Alzheimer's disease has such a long development period, patients can benefit from frequent testing and receive early treatment. However, due to their high cost and limited choice, current clinical diagnostic imaging techniques do not match the specific needs of screening methods [3, 4]. We made it a priority in this study to assess the retinal, particularly the retinal vasculature, as a potential solution for performing dementia assessments in Alzheimer's chronic conditions. Inflammatory alterations may begin 20+ years before neurological dysfunction manifests, and though the time neurotoxic effects manifest, cerebral deterioration has so far gradually extended. The Alzheimer's Society, the National Institute of Health, and thus the Global Advisory Committee on AD have suggested a study paradigm given a set of confirmed indicators connected towards both kinds of abnormalities that are proxies for AD to identify AD in actual persons [5–7]. All across the process, flexible scalable neural nets were used. The process obtained an overall accuracy rate of 82.44 percent using data from either the UK Biobank. It included a saliency analysis of this pipeline's understandability in addition to a high classifier shown in Figure 1. The detection of transgenic mice is carried out from the input fundus image, but the existing approaches possess a higher false detection rate which degrades the accuracy of the system. Additionally, the following problems are faced in optimal detection of classes which are listed as

- Difficulty in feature differentiation: the detection of transgenic mice is based on various features such as texture, color, and intensity, but the differentiation of these minute features from each other is a hard task that degrades the computation of accurate diseases
- Class overlapping: the class of input image is also determined by the existing approaches, but the limited set of training data of each severity results in a class imbalance problem affecting the accuracy of classification
- Improper preprocessing: the execution of conventional proper preprocessing and effective enhancement of contrast techniques by the existing approaches results in difficulty in identifying the features from the background

The major objective of this study is to provide precise classification between the WT and TMM and to compute the accuracy of the diseases in an accurate manner. This objective is achieved by fulfilling the subobjectives which are listed as follows:

- To minimize the level of artifacts in the input image by performing effective preprocessing of the image
- To maximize the precise identification of features from the preprocessed image by performing enhancement of contrast level
- To effective classify the images into two classes based on the extraction of significant features
- To determine the features related to the disease based on the variation in the intensity of the features for the purpose of diagnosis

## 2. Related Work

Under [8] article, authors investigated alterations in optic disc linked with Alzheimer's disease using the retinal as a window into the central and peripheral nervous system. Optical coherence tomography would be used to analyse the retinas of transgenic mice models (TMM) and wild-type (WT) of Alzheimer's disease, and support vector machines with the radial basis function kernel were used to categorize the cells in the retina into TMM and WT classes. At the age of four months, predictions were over 80% accurate, and at the age of eight months, they were over 90% accurate. In line with the results, feature extraction of generated fundus images acquired shows a much more diverse retinal architecture in mouse models at the age of eight.

Utilizing coregistered angle-resolved [9] low-coherence interferometry (a/LCI) and optical coherence tomography, we obtained insight light scattering data from the retinas of triple transgenic Alzheimer's disease (3xTg-AD) mice and wild-type (WT) age-matched controls (OCT). Visual guiding and segmentation depths supplied by cross OCT B-scans were used to obtain perspective dispersion data from the peripheral nerve layer, outer papillary overlay, and endodermal epithelial. When comparing vivo mouse cells in the retina to WT controls, OCT imaging revealed a substantial weakening of the nerve fibre layer. The a/LCI scattering measures offered additional information which helps to differentiate AD mice by quantifying tissue heterogeneity. While compared to the WT mice, the AD mice's eyes demonstrated an increased range of values in motor neuron layer interferometric strength.

In [10], the authors of this article describe the relationship between retinal image characteristics and cerebral-amyloid (A) load in the hopes of establishing a benign method for predicting A deposit in Alzheimer's illness. Moreover, while comparing to A+ individuals, a substantial variation in textural predefined sequence across retina capillaries and their neighbouring areas was detected in A+ participants. Using the collected characteristics, classifiers are trained to classify new individuals. Including an efficiency of 85 percent, the classification can distinguish A+ patients from “A” patients.

## 3. Proposed Work

This section presents the description of the proposed model for the classification of transgenic mice using SVMs.

### 3.1. Preprocessing

For enhancing the information for the disease diagnosis system, it is necessary to use some of the preprocessing steps as follows:

(i) Artifacts removal: blurriness, poor edges, and illumination are called as artefacts, which are removed using the nonlinear diffusion filtering algorithm, which eliminates all kinds of artefacts and ensures the image quality in terms of illumination correction and edge preservation
(ii) Contrast enhancement: low contrast is one of the important issues of image classification. In this work, we consider that contrast enhancement is an optimization problem that intention is to optimize the pixel values based on the contrasting level of the input image.
(iii) Image normalization: normalization of the image is valuable to variation of pixel intensity or RGB color values for retina images that increase the quality of acquired fundus images by decreasing the equipment and desired noises of the retina images. Following, the misrepresentations and fluctuations that happened in the retina images because of inexact image internment are recognized. Throughout the normalization of the image, the learned image is transformed into predetermined values. The formula for the estimation of image normalization is exactly denoted as follows. Image normalization is a technique of preprocessing that uses certain types of range as an expected outcome for the given inputs. It is useful for the prediction of forecasting purposes. Here, we know that there are several ways for forecasting and also prediction to maintain the large variations and also forecasting the normalized values makes the closer. There are some existing normalization techniques that are used for image normalization, which are as follows:
  - Min-max normalization
  - Z-score normalization
  - Decimal scaling

**Figure 2:**
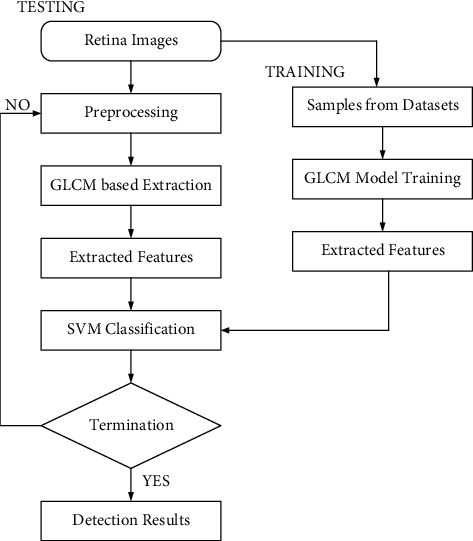
Proposed work.

Figure 2 describes the proposed work. In the following, the description of these normalization techniques is given in detail.

(i) Min-max normalization: this technique provides the transformation function for linear cases by the original values of data which is known as the min-max normalization technique. This technique uses predefined boundary for the specific retina images. The min-max normalization for the proposed technique is estimated as follows:
  (1) $$\begin{array}{c} \hat{A}=\;\left(\frac{A-\;\min_{A}}{\max_{A}-\min_{A}}\right)\times \left(D-C\right)+C, \end{array}$$
    where $\hat{A}$ represents the normalized value of min-max data values and when the predefined boundary is between the C and D. When the range of values of A and B is matched between one another is used for result validation.
(ii) In general unstructured data can be normalized using Z-score normalization, which is represented as follows:
  (2) $$\begin{array}{c} V_{i}^{\prime }=\;\frac{V_{i}-E}{STD\;\left(E\right)}, \end{array}$$
   where *V*_*i*_ is the *Z* − *score* normalized values of the input, and  *E* represents the row *E* of the ith column.
  (3) $$\begin{array}{c} STD\;\left(E\right)=\;\sqrt{\frac{1}{\left(n-1\right)}\overset{n}{\underset{i=1}{\sum }}{\left(v_{i}-E\right)}^{2}}, \end{array}$$
  (4) $$\begin{array}{c} E=\;\frac{1}{\left(n\right)}\overset{n}{\underset{i=1}{\sum }}{\left(v_{i}-E\right)}^{2}, \end{array}$$
   where  *E* is the mean value of the inputs. This technique uses five rows such as *X*,  *Y*,  *Z*,  *U*, and V for different columns for “*N*” for each row in which each row represents the Z-score technique that applies for computation of the normalized values. So that the standard deviation of the row is equal to the zero, then all values for the row are fixed to the zero values. It also gives the range of values between 0 and 1. In the technique of decimal scaling, the range is between −1 and 1. Based on the decimal scaling for image normalization, it is computed by the following equation:
  (5) $$\begin{array}{c} v^{i}=\;\frac{v}{10^{j}}, \end{array}$$
where *v*^*i*^ represents the scaled values, *v* represents the range of values, and *j* represents the small integer Max (*v*_*i*_) < 1. The above-mentioned techniques can be useful for discussing the values of normalization.

The combination of the above three techniques helps in producing the result, that is, improved min-max decimal with Z_normalization). The proposed retina image normalization technique is the advanced and most effective normalization technique that uses various types of input images, and also, it produces outputs in the range of 0 to 1. The normalization techniques can be possible for taking the average values as a threshold and then normalizing or replacing the values of the other side of pixels using the mean and standard deviation.

As compared to the min-max, Z-score, and decimal scaling techniques for image normalization, the proposed advanced technique for image normalization produces an effective result. The proposed technique is used for image normalization that produces the following advantages than the other existing methods.

- Suited for any volume of datasets (large, small, or medium size datasets)
- Individual pixel-based scaling and transformation are possible
- Used to make the independent data size
- Set the range between 0 and 1 and have the normalized values
- Easy to apply for whole numerical data values

The proposed innovative normalization technique is mathematically expressed as follows:

(6) $$\begin{array}{c} Y=\;\frac{\left(\left|X\right|-\;\left(10^{n-1}\right)\times \left(\left|A\right|\right)\right)}{10^{n-1}}, \end{array}$$

where *X* represents the particular element of the data, *N* represents the number of digits in the element of *X*, *A* represents the pixel element for 1st digit *X*, and *Y* represents the scaled 1 value between 0 and 1. The proposed model is applicable for all types of input lengths to the full types of integers. This technique is different than the existing normalization approaches which are as follows:

- Changed from the unstructured to the structured one.
- Purpose of formulation/scaling.
- All the inputs are numerical data only.
- Low light enhancement: recent methods for low light enhancement methods are not assured for applying in low light environments. In order to design the new method for low light enhancement, it should focus on the following.
- Enhance the efficiency and robustness of the low-light image enhancement algorithms, and the previous methods are not supported for insufficient techniques to meet the needs of current applications.
- This method should be able to adjust for the different types of images on different scales to produce an extraordinary result.
- Minimize the complexity (time, space) for overall computations that are available to all the methods. This satisfies the practical application, and also, real-time images must be supported to use this.
- Most of the existing techniques are used for longer operations and hence take more processing time. And still, it leads to two problems such as detail ambiguity and color deviations.
- Establishes the higher quality of the image evaluation in which image information recovery and color recovery functions are used for adjusting the low light enhancements.

To address these issues for this step, multiscale Retinex theory is proposed, which is a color restoration method that processes the image quality for further enhancement using the single-scale Retinex or multiscale Retinex method. This algorithm is applied for 3 kinds of color channels such as *R*, *G*, and *B* separately. Thus, here, the original image is converted into the number of channels. This avoids the color distortion issue. For each algorithm, the color recovery factor *C* is computed. This computes the proportional relationship between the *R*, *G*, and *B* channels. This mathematically expressed equation is as follows:

(7) $$\begin{array}{c} C_{i}\left(x,y\right)=\;F_{i}\frac{I_{i}\left(x,y\right)}{\sum_{i}^{3}I_{i}\left(x,y\right)}, \end{array}$$

where *F* represents the function for mapping the color values, and the performance of the best color intensity values for restoration and recovery helps in mapping the function in which logarithmic is used for computations of color recovery.

(8) $$\begin{array}{c} C_{i}\left(x,y\right)=\beta \times \;\log\left(\alpha \right)\frac{I_{i}\left(x,y\right)}{\sum_{i}^{3}I_{i}\left(x,y\right)}, \end{array}$$

where *α* and *β* are the mathematical expressions for variables in which logarithmic function is computed and rewritten as follows:

(9) $$\begin{array}{c} MRT=\log\;R_{i}\left(x,y\right), \end{array}$$

(10) $$\begin{array}{c} \overset{k=1}{\underset{N}{\sum }}C_{i}w_{k}\left\{\log\;I_{i}\left(x,y\right)-\log\left[G_{k}\left(x,y\right)\times I_{i}\;\left(x,y\right)\right]\right\}. \end{array}$$

This algorithm considers the merits of the convolution operation using Gaussian computations. For the multiscale, that is, small, medium, and large range of patches yield good ideal effects. The performance of color restoration is improved using the color recovery factor values since it is updated for concurrent iterations.

### 3.2. Feature Extraction

The gray level co-occurrence matrix captures numerical features of a texture using spatial relations of similar gray tones. The following are the features derivable from a normalized co-occurrence matrix in Table 1.

#### 3.2.1. Computation of Textural Features from Normalized GLCM

Energy: measures the uniformity (or orderliness) of the gray level distribution of the image
Range = [0 1]
  (11) $$\begin{array}{c} \sum_{i,j}p\left(i,j\right)=0.166^{2}+0.083^{2}+0.042^{2}+0.083^{2}+\\ 0.166^{2}+0+0.042^{2}+0+0.0250^{2}+0.042^{2}. \end{array}$$
Homogeneity: measures the smoothness (homogeneity) of the gray level distribution of the image; range = [0 1]
  (12) $$\begin{array}{c} \sum_{i,j}\frac{p\left(i,j\right)}{1+\left|i-j\right|}=0.8155. \end{array}$$
Contrast: Tables 2 and 3 give a measure of the intensity contrast between a pixel and its neighbor over the whole image
Range = [0 (size(GLCM,1)-1)2]

### 3.3. Classification

**Figure 3:**
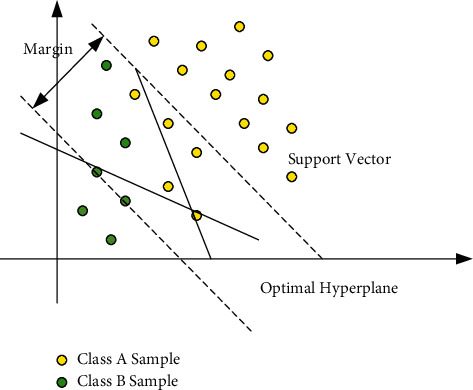
SVM for classification of transgenic mice.

For the classification of retinal images into two classes such as WT and TMM, SVMs are used, and the optimum kernel function is selected from the set of kernel functions for the classifications.  Figure 3 discusses the pictorial representation for the classification using SVMs.

## 4. Experimental Results and Discussion

This proposed work is mainly implemented to provide precise classification between the WT and TMM for obtained accuracy of the diseases in an accurate manner. This proposed work undergoes preprocessing and feature extraction. The preprocessing technique is performed to minimize the level of the artifacts in the input image, and the enhancement of the contrast level is performed to increase the precise identification of features from the preprocessed image. Then, feature extraction is implemented to classify the images into two classes based on the extraction of significant features in an effective manner.

**Figure 4:**
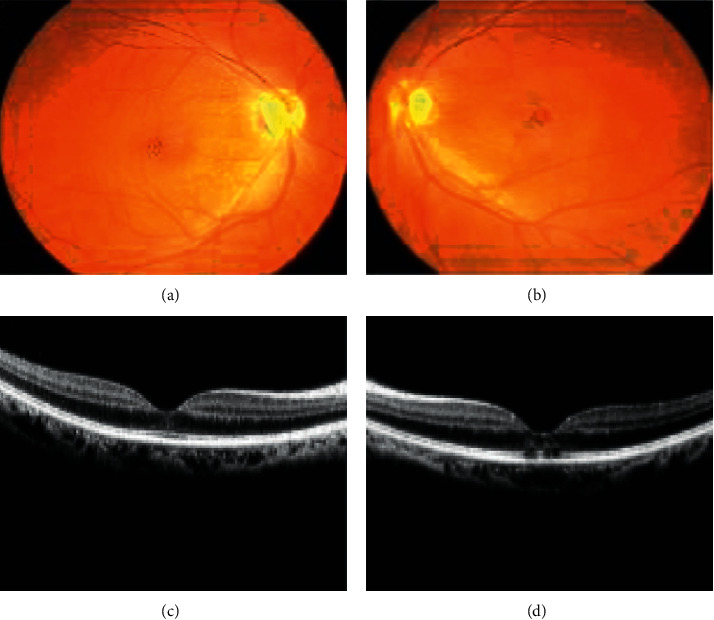
(a) and (b) retinal images, and (c), (d) OCT images.

In this section, the performance of the proposed model is implemented for the sum of images in the dataset in Figure 4.  Table 4 describes the confusion matrix for the two classes with the use of four kinds of metrics. The definition of each metric is given below, and classifier performance is shown in Table 5.

- True positive (TP) is the no. of candidates correctly identified as TMM
- False positive (FP) is the no. of candidates incorrectly identified as TMM
- True negative (TN) is the no. of candidates correctly identified as non-TMM
- False negative (FN) is the no. of candidates incorrectly identified as non-TM

(13) $$\begin{array}{c} \text{Sensitivity}=\frac{TP}{TP+FN}=\frac{18}{18+06}=75\mathrm{\%,} \end{array}$$

(14) $$\begin{array}{c} \text{Specificity}=\frac{TP}{TP+FN}=\frac{1643}{2256}=72.82\mathrm{\%,} \end{array}$$

(15) $$\begin{array}{c} FPR=\left(1-\text{Specificity}\right)=\frac{TP}{TP+FN}=\frac{613}{2256}=0.271, \end{array}$$

(16) $$\begin{array}{c} \text{Accuracy}=\frac{TP+TN}{TP+FN+TN+FP}=\frac{1661}{2280}=72.85\%. \end{array}$$

## 5. Conclusion

Alzheimer's disease is a progressive neurodegenerative illness defined by the presence of Amyloid–(A) in the brain. Nevertheless, because the degenerative concerns of the brain are complicated in classification, precise detection of this condition is a difficult process. The abnormalities in retinal fundus images for Alzheimer's disease are divided into two categories in this paper: wild-type (WT) and transgenic mice model (TMM). Optical coherence tomography (OCT) pictures are utilised to classify the patients into 2 categories for assessment. SVMs are used to classify the data, only with the best kernel selected via an evolutionary method. The RBF kernel function outperforms the other SVM support vectors in terms of accuracy. The textural properties of retinal fundus images are used to deal with just an efficient categorization utilising SVM. The overall accuracy reached 92% and 91% of precision for the classification of transgenic mice.

## Figures and Tables

**Table 1:** Statistical GLCM features-22.

Feature name	Description
Autocorrelation	Sum of squares
Contrast	Sum average
Correlation 1 & 2	Sum variance
Cluster prominence	Sum entropy
Cluster shade	Difference variance
Dissimilarity	Difference entropy
Energy	Information measure of correlation 1 & 2
Entropy	Inverse difference normalized (INN)
Homogeneity 1 & 2	Inverse difference moment normalized
Maximum probability	

**Table 2:** List of features.

S.No	Feature	Formula	Description
1	Autocorrelation	∑_*i*,*j*_(*ij*)*p*(*i*, *j*)	It measures the coarseness of an image and evaluates the linear spatial relationships between texture primitives.
2	Contrast	∑_*i*,*j*_|*i* − *j*|^2^*p*(*i*, *j*)	Represents the amount of local gray level variation in an image; a high value of this parameter may indicate the presence of edges, noise, or wrinkled textures in the image.
3	Correlation 1	∑_*i*,*j*_(*j* − *μ*_*y*_)*p*(*i*, *j*)/*σ*_*x*_*σ*_*y*_	Gives a measure of how correlated a pixel is to its neighbor over the whole image.
4	Correlation 2	∑_*i*,*j*_(*ij*)*p*(*i*, *j*) − *μ*_*x*_*μ*_*y*_/*σ*_*x*_*σ*_*y*_	Gives a measure of gray level linear dependence between the pixels at the specified positions relative to each other.
5	Cluster shade	∑_*i*,*j*_(*i*+*j* − *μ*_*x*_ − *μ*_*y*_)^3^*p*(*i*, *j*)	Cluster shade and cluster prominence are measures of the skewness of the matrix, in other words the lack of symmetry.
6	Cluster prominence	∑_*i*,*j*_(*i*+*j* − *μ*_*x*_ − *μ*_*y*_)^4^*p*(*i*, *j*)	Gives a measure of local intensity variation.
7	Dissimilarity	∑_*i*,*j*_|*i* − *j*|*p*(*i*, *j*)	Dissimilarity measure belongs to the contrast group of texture metrics. Gives a measure of dissimilarity.
8	Energy	∑_*i*,*j*_*p*(*i*, *j*)^2^	Measures the uniformity (or orderliness) of the gray level distribution of the image; images with a smaller number of gray levels have larger uniformity.
9	Entropy	−∑_*i*,*j*_*p*(*i*, *j*)log(*p*(*i*, *j*))	Inhomogeneous images have a low entropy, while a homogeneous scene has high entropy.
10	Homogeneity 1	∑_*i*,*j*_*p*(*i*, *j*)/1+|*i* − *j*|	Gives a value that measures the closeness of the distribution of elements in the GLCM to the GLCM diagonal.
11	Homogeneity 2	∑_*i*,*j*_1/1+(*i* − *j*)^2^*p*(*i*, *j*)	Measures the smoothness (homogeneity) of the gray 12level distribution of the image; it is inversely correlated with contrast—if contrast is small, usually homogeneity is large.
12	Maximum probability	$\text{MAX}\underset{i,j}{p}\left(i,j\right)$	Gives a measure of max. Frequency of occurrence of pixel pairs.
13	Sum of squares: Variance	∑_*i*,*j*_(*i* − *μ*)^2^*p*(*i*, *j*)	Measures the dispersion (with regard to the mean) of the gray level distribution.
14	Sum average	∑_*i*−2_^2*Ng*^*ip*_*x*+*y*_(*i*)	Measures the mean of the gray level sum distribution of the image.
15	Sum variance	∑_*i*−2_^2*Ng*^(*i* − [∑_*i*−2_^2*Ng*^*ip*_*x*+*y*_(*i*)])^2^	Measures the dispersion (with regard to the mean) of the gray level sum distribution of the image.
16	Sum entropy	−∑_*i*−2_^2*Ng*^*p*_*x*+*y*_(*i*)log{*p*_*x*+*y*_(*i*)}	Measures the disorder related to the gray level sum distribution of the image.
17	Difference variance	${\sum_{i-2}^{2Ng}\left(i-\left[\sum_{i-2}^{2Ng}ip_{x-y}\left(i\right)\right]\right)}^{2}$	Measures the dispersion (with regard to the mean) of the gray level difference distribution of the image.
18	Difference entropy	−∑_*i*−2_^2*Ng*^*p*_*x*−*y*_(*i*)log{*p*_*x*−*y*_(*i*)}	Measures the disorder related to the gray level difference distribution of the image.
19	Information measure of correlation 1	*HXY* − *HXY*1/max{*HX*, *HY*}	H is the entropy. *HXY*1=−∑_*i*,*j*_*p*(*i*, *j*)log(*p*_*x*_(*i*), *p*_*y*_(*j*)).
20	Information measure of correlation 2	$\sqrt{1-e^{\left[-2\left(HXY2-HXY\right)\right]}}$	*HXY*2=−∑_*i*,*j*_*p*(*i*, *j*)*p*_*y*_(*j*)log(*p*_*x*_(*i*), *p*_*y*_(*j*)).
21	Inverse difference normalized (IDN)	∑_*i*=0_^*Ng*−1^*p*(*i*, *j*)/1+(|*i* − *j*|/*N*)	IDMN and IDN measure image homogeneity as it assumes larger values for smaller gray tone differences in pair elements. It is more sensitive to the presence of near diagonal elements in the GLCM. It has maximum value when all elements in the image are same.
22	Inverse difference moment normalized (IDMN)	∑_*i*=0_^*Ng*−1^*p*(*i*, *j*)/1+(|*i* − *j*|^2^/*N*^2^)	

**Table 3:** List of shape features.

Sl.No	Feature	Formula	Description
1	Circularity	*C*=4*π*Area/(Perimeter)^2^	A measure of roundness or circularity (area-to-perimeter ratio) can be obtained as the ratio of the area of an object to the area of a circle with the same convex perimeter.1-for a circular object and <1 or >1 for an object that departs from circularity.
2	Eccentricity	*E*=axislength_short_/axislength_long_	Eccentricity is the ratio of the length of the short (minor) axis to the length of the long (major) axis of an object. Range: 0 to 1.
3	Orientation	*θ*=1/2tan^−1^(2*μ*_11_/*μ*_20_ − *μ*_02_)	The orientation is the angle between the horizontal line and the major axis. It indicates the overall direction of the shape. Range: −90° to 90°

**Table 4:** Confusion matrix.

Total candidates (2280)		True class	
		Positive	Negative
Predicted class	Positive	TP (18)	FP (613)	TP + FP (631)
	Negative	FN (06)	TN (1643)	TN + FN (1647)
		TP + FN (24)	TN + FP (2256)	

**Table 5:** Classifier performance.

Classifier	Accuracy (%)	Sensitivity (%)	Specificity (%)
Decision tree	96	94	97
Neural network	74	98	73
Random forest	98	65	98
SVM	99	98	99

## Data Availability

The datasets used and/or analyzed during the current study are available from the corresponding author upon reasonable request.
